# Effects of Dual-Frequency Ultrasound-Assisted Thawing Technology on Thawing Rate, Quality Properties, and Microstructure of Large Yellow Croaker (*Pseudosciaena crocea*)

**DOI:** 10.3390/foods11020226

**Published:** 2022-01-14

**Authors:** Hao Cheng, Chuhan Bian, Yuanming Chu, Jun Mei, Jing Xie

**Affiliations:** 1 College of Food Science and Technology, Shanghai Ocean University, Shanghai 201306, China; m200300836@st.shou.edu.cn (H.C.); m200300826@st.shou.edu.cn (C.B.); m190300744@st.shou.edu.cn (Y.C.); jmei@shou.edu.cn (J.M.); 2 National Experimental Teaching Demonstration Center for Food Science and Engineering, Shanghai Ocean University, Shanghai 201306, China; 3 Shanghai Engineering Research Center of Aquatic Product Processing and Preservation, Shanghai 201306, China; 4 Shanghai Professional Technology Service Platform on Cold Chain Equipment Performance and Energy Saving Evaluation, Shanghai 201306, China

**Keywords:** thawing rate, ultrasound-assisted thawing, quality properties, microstructure, large yellow croaker

## Abstract

This research evaluated the effects of dual-frequency ultrasound-assisted thawing (UAT) on the thawing time, physicochemical quality, water-holding capacity (WHC), microstructure, and moisture migration and distribution of large yellow croaker. Water thawing (WT), refrigerated thawing (RT), and UAT (single-frequency: 28 kHz (SUAT-28), single-frequency: 40 kHz (SUAT-40), dual-frequency: 28 kHz and 40 kHz (DUAT-28/40)) were used in the current research. Among them, the DUAT-28/40 treatment had the shortest thawing time, and ultrasound significantly improved the thawing rate. It also retained a better performance from the samples, such as color, texture, water-holding capacity and water distribution, and inhibited disruption of the microstructure. In addition, a quality property analysis showed that the pH, total volatile basic nitrogen (TVB-N), and K value were the most desirable under the DUAT-28/40 treatment, as well as this being best for the flavor of the samples. Therefore, DUAT-28/40 treatment could be a possible thawing method because it improves the thawing rate and maintains the quality properties of large yellow croaker.

## 1. Introduction

Large yellow croaker (*Pseudosciaena crocea*) is rich in lipids and protein and widely cultured in China [1]. Due to the high moisture content, nutrient composition, and enzyme activity of large yellow croaker, it is highly vulnerable to contamination by pathogenic bacteria, which causes lipid oxidation, protein denaturation, and texture changes, resulting in a loss of commercial value [2,3]. To solve these problems, low-temperature frozen storage is usually applied. However, the quality of frozen products is influenced by both the freezing and thawing processes, especially the thawing process [4]. Thawing is an indispensable part of the production and processing of aquatic products. Unsuitable thawing methods may lead to quality loss [5]. Research on new thawing methods is currently in demand for the efficient utilization of aquatic products.

Ultrasound-assisted thawing (UAT) has been widely studied and applied in recent years. Studies have shown that UAT can reduce the thawing time and maintain the quality of frozen food [4,5]. UAT could also speed up the thawing rate and maintain the quality of fish, in aspects such as WHC (water-holding capacity) and texture [6]. Guo et al. reported that UAT at 400 W could better improve the thawing process efficiency and quality of frozen foods [7]. Moreover, under UAT (power ultrasound intensity: 2.39, 6.23, 11.32, and 20.96 W cm^−2^; treatment time: 30, 60, 90, and 120 min), the gel properties and WHC of proteins were also improved [8]. The advantages of using ultrasound-assisted thawing with the right frequency and power were decreased thawing time, more uniform thawing temperature of the products, and better impact on the myofibrillar protein gel formation compared with other traditional thawing methods [9]. Li et al. [10] reported that ultrasound-assisted thawing (20 °C, 45 min) of frozen porcine longissimus muscle had reduced the damage to conformational change and gel formation of myofibrillar protein when compared with water thawing. In addition, Chen et al. [11] found that ultrasound-assisted thawing could save 13.5–40.4% in thawing time, and high ultrasonic frequency (80 kHz and 100 kHz) did not cause high thawing loss. Thawing at higher ultrasonic frequency could reduce the damage of the secondary and tertiary structure of myofibrillar proteins. Recently, Bian et al. [12] reported that multifrequency UAT significantly reduced the thawing time of frozen samples and maintained better quality attributes in comparison with SUAT-20 kHz, DUAT-20/28 kHz, and TUAT-20/28/40 kHz. It similarly focused on the multifrequency effect: what distinguished our study was that only two different single-frequency treatments were applied to explore the different effects of the two frequencies in combination and in isolation. Wang et al. [13] studied the effect of sweep frequency ultrasound thawing (SFUT) on gel, and the experiment was set with fluctuating frequency instead of constant frequency. It mainly paid attention to effects on gel, and SFUT improved gel properties, as shown in the study. In addition, it was found that DUAT-20/40 kHz had more desirable behavior than SUAT-20 kHz and TUAT-20/40/60 kHz [14], and it also focused on the gel. Based on this research, it was determined that DUAT performed better than SUAT and TUAT. It was considered worth exploring the different effects of two frequencies in combination and in isolation. The effects on quality under UAT were the focus of the study.

Frequency is an important parameter of ultrasound and poses no potential danger. The use of different frequencies can effectively improve the thawing process as described in the above research. There are few studies on UAT methods at different frequencies. Therefore, in this study, three types of UAT (SUAT-28, SUAT-40, and DUAT-28/40; ultrasonic power: 200 W) were used to study the effects of UAT on the quality of large yellow croaker, especially its water-holding capacity, water distribution and migration, K value, TVB-N, and the microstructure of the muscle. The study mainly focused on the effects of ultrasound-assisted thawing under different frequencies.

## 2. Materials and Methods

### 2.1. Preparation of Fish Samples

**Figure 1:**
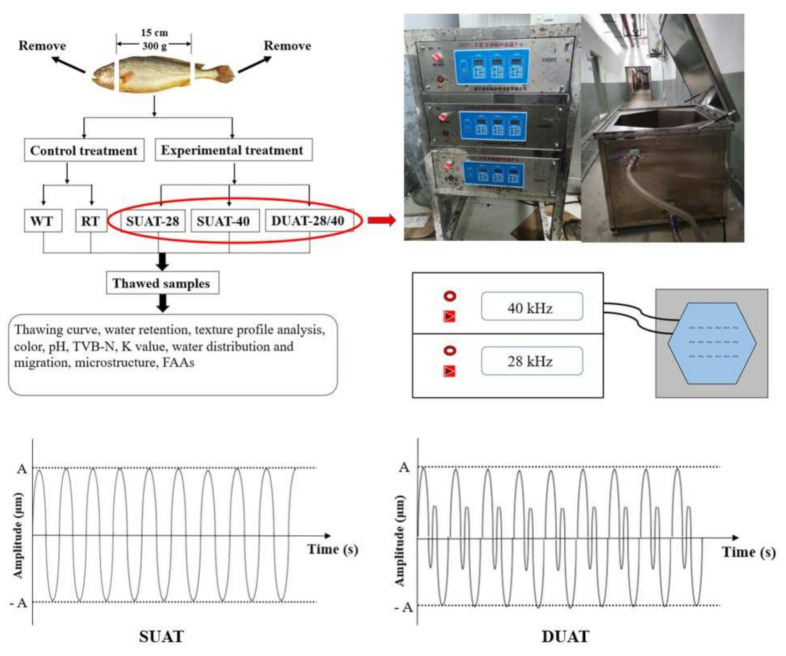
Diagram and possible mechanisms of the ultrasonic thawing system.

Large yellow croaker was acquired from Luchao Harbor Market and kept alive for prompt movement back to the laboratory. After being killed quickly, the fish were moved to a spiral freezer (Yantai Moon Co., Ltd., Yantai, Shandong, China) at a temperature of −35 °C and a wind velocity of 10 m/s for freezing for about half an hour. In order to facilitate further sample taking and measurement of experimental indicators, the heads and tips of the fish were all removed. Then the fish were quickly transferred to a −20 °C refrigerator and stored for three days. After that, the frozen samples were thawed using WT (water thawing), RT (refrigerator thawing), SUAT-28 (single-frequency ultrasound-assisted thawing at 28 kHz), SUAT-40 (single-frequency ultrasound-assisted thawing at 40 kHz), and DUAT-28/40 (dual-frequency ultrasound-assisted thawing at 28 kHz and 40 kHz). This was finished when the internal temperature of the fish reached 4 °C on the temperature monitor. WT and RT were used as control treatments, and SUAT-28, SUAT-40, and DUAT-28/40 were used as experiment treatments (Figure 1). The treatments were conducted at room temperature and RT was conducted in the refrigerator at 4 °C. They were considered finished when the internal temperature reached 4 °C. The power of all ultrasound-assisted thawing was 200 W.

Figure 1 shows the main research content and ultrasonic-assisted thawing device used in the study. The device consisted of a control panel and sink. The control panel was used to monitor power and frequency, and fish were thawed in the sink. The possible mechanisms of ultrasound-assisted thawing under different frequencies were also proposed to account for the more uniform impact from ultrasound.

### 2.2. Thawing Curve

**Figure 2:**
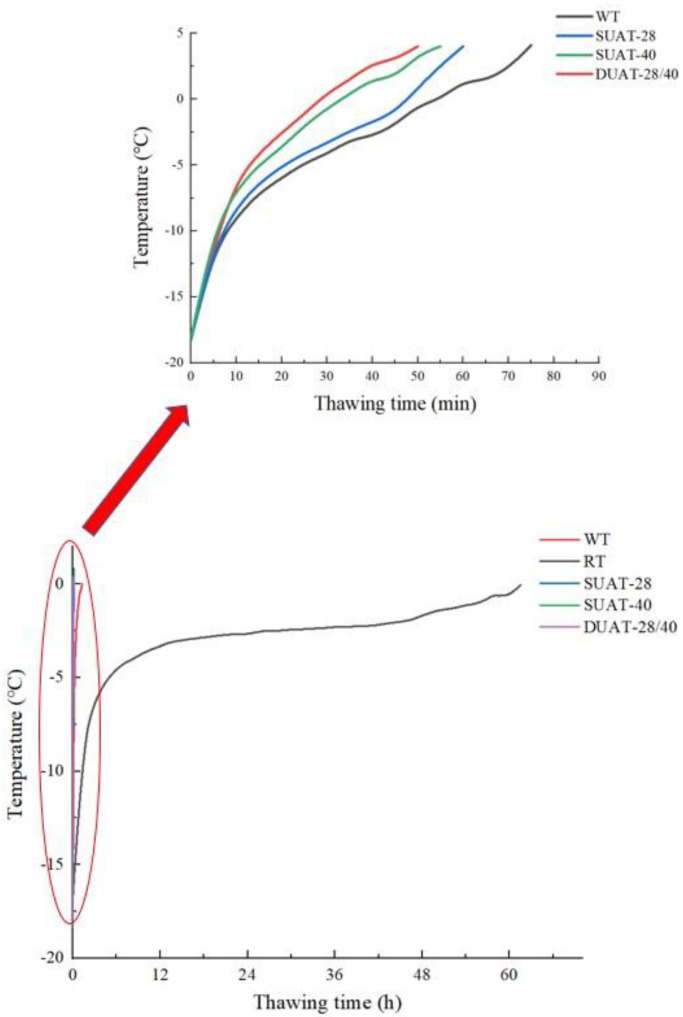
Differences in thawing curve of large yellow croaker under different thawing methods. (WT: water thawing; RT: refrigerated thawing; SUAT-28: single-frequency ultrasound-assisted thawing at 28 kHz; SUAT-40: single-frequency ultrasound-assisted thawing at 40 kHz; DUAT-28/40: dual-frequency ultrasound-assisted thawing at 28 kHz and 40 kHz).

The temperature change was monitored by inserting the sensor of a multiple-point thermometer (Fluke 2640A, Everett, WA, USA) into the muscle center of the large yellow croaker. For each thawing method, three temperature-recording spots were set up, and the most appropriate thawing curve was selected, as shown in Figure 2. Each measurement was taken in triplicate.

### 2.3. Determination of Water Retention

#### 2.3.1. Thawing Loss

The water on the surface of the thawed fish was wiped, and the weight was measured as *W*_1_ [15]. *W*_0_ meant the weight of fish before thawing. This is shown in Equation (1). Only one trial was conducted per experiment.

(1) $$\mathrm{Thawing}\;\mathrm{loss}\;\%=\frac{W_{0}-W_{1}}{W_{0}}\times 100\%$$

where *W*_0_ and *W*_1_ refer to the weight before and after thawing.

#### 2.3.2. Water-Holding Capacity

After defrosting the fish, the water on the surface of the fish was wiped, and 2.00 g of the sample was weighed. Centrifugation was conducted at 5000× *g* at 4 °C for 10 min [16]. The water-holding capacity was then calculated based on the formula below. The weight before centrifugation was called *M*_1_, and the weight after centrifugation was called *M*_2_, as shown in Equation (2). Each measurement was taken in triplicate.

(2) $$\mathrm{Water-holding}\;\mathrm{capacity}\;\left(\%\right)\;=\frac{M_{2}}{M_{1}}\times 100\%$$

where *M*_1_ and *M*_2_ refer to the weight before and after centrifugation.

#### 2.3.3. Cooking Loss

5.00 g of fish samples were weighed as *W*_1_ and cooked in a water bath at 85 °C for 10 min. Then the weight was measured after cooking, denoted as *W*_2_ [17]. Each measurement was taken in triplicate. It was calculated using Equation (3).

(3) $$\mathrm{Cooking}\;\mathrm{loss}\;\%=\frac{W_{1}-W_{2}}{W_{1}}\times 100\%$$

where *W*_1_ and *W*_2_ refer to the weight before and after cooking.

### 2.4. Determination of the Texture Profile Analysis (TPA)

The samples were cut into 20 mm × 15 mm × 10 mm pieces, and the texture properties were analyzed using a texture analyzer [6]. The analysis software name was TA.XTplusC Texture Analyzer (Stable Micro Systems, Ltd., Godalming, Surrey, UK). The pre-test, test, and post-test speeds were 3, 1, and 1 mm/s, respectively, with a probe number (code) of Auto-5 g and a trigger force of 5 g. Each measurement was taken in triplicate.

### 2.5. Determination of Color

The samples were placed on a flat plate, and the ΔL* (brightness/darkness), Δa* (redness/greenness), and Δb* (yellowness/blueness) values were calculated using a colorimeter (CR-400, Konica Minolta, Tokyo, Japan). Changes in color were calculated as Δ*E* using Equation (4). Each measurement was taken in triplicate.

(4) $$△E=\sqrt{{\left(△L^{*}\right)}^{2}+{\left(△a^{*}\right)}^{2}+{\left(△b^{*}\right)}^{2}}$$

where ΔL*, Δa*, Δb* refer to the value of brightness/darkness, redness/greenness, and yellowness/blueness after calibration. In addition, ΔL*, Δa*, and Δb* were the differences between L*, a*, and b* values before samples were UAT-treated and after samples were UAT-treated, respectively [18].

### 2.6. Determination of the pH Value

According to the method of Lv et al. [19], 2.00 g of the chopped fish samples were mixed with 20 mL of physiological saline (0.9%, *m*/*v*) and centrifuged at 1000× *g* for 5 min at 4 °C. The pH value was measured using a pH meter (Mettler Toledo, Greifensee, Switzerland). Each measurement was taken in triplicate.

### 2.7. Determination of TVB-N

The fish samples were minced and weighed to approximately 5 g for measurement. The TVB-N was calculated via microtitration and shown as mg N/100 g of the fish samples [20]. Each measurement was taken in triplicate.

### 2.8. Determination of the K Value

A 2.00 g sample was removed and homogenized with a 10% (*v*/*v*) perchloric acid solution. The sample was centrifuged at 8000× *g* for 15 min at 4 °C, and the precipitate was mixed with a 5% (*v*/*v*) perchloric acid solution and centrifuged under the same conditions. The supernatants were combined, and the pH was adjusted to 6.5 with the phosphoric acid. The mixture was filtered through a 0.22 μm disposable filter, and 1 mL of the mixture was extracted [21]. The K value was then analyzed using the High-Performance Liquid Chromatography (HPLC) (Waters 2695, Milford, MA, USA) procedure suggested by Li and Zhan [22]. Each measurement was taken in triplicate.

The HPLC system with a Diamonsil-C18 column (250 × 4.6 mm) and a 254 nm UV detector was used to quantify adenosine triphosphate (ATP) and its catabolites including adenosine diphosphate (*ADP*), adenosine monophosphate (*AMP*), inosine monophosphate hypoxanthine (*IMP*), riboside (*HxR*), and hypoxanthine (*Hx*). Mobile phase A (phosphate) and mobile phase B (methanol) were subjected to gradient elution using the following procedure: 0 min to 20 min, 95% A and 5% B. The flow rate was 1 mL/min, the column temperature was 30 °C, and the injection volume was 10 μL. The K value was calculated using Equation (5):

(5) $$K\;value\;\left(\%\right)\;=\frac{HxR+Hx}{ATP+ADP+AMP+IMP+HxR+Hx}\times 100$$

where *ADP, AMP, ATP, IMP, Hx*, and *HxR* refer to adenosine diphosphate, adenosine monophosphate, adenosine triphosphate, inosine monophosphate hypoxanthine, hypoxanthine, and riboside, separately.

### 2.9. Water Distribution and Migration

The water distribution and migration within the fish were assessed using a low-field nuclear magnetic resonance (LF-NMR) analyzer (MesoMR23-060 H.I, NiuMeng, Shanghai, China). The specific settings were as follows: receiver bandwidth frequency (SW) = 100 kHz, SF = 21 MHz, RFD = 0.08 ms, RG1 = 20 db, P1 = 19 us, DRG1 = 6, TD = 400,100, DR = 1, TW = 2000 ms, NS = 4, P2 = 37 us, TE = 0.5 ms, and NECH = 8000 [23]. Each measurement was taken in triplicate.

### 2.10. Scanning Electron Microscope (SEM)

The microstructure was observed using an SEM (Hitachi HT 7700, Tokyo, Japan) as proposed by Cao et al. [24]. The samples were cut into 3 mm × 3 mm × 3 mm pieces, fixed with a 2.5% glutaraldehyde solution for 24 h, and then washed with distilled water to resist the fixer deposits. The samples were dewatered under an alcohol gradient (30%, 50%, 70%, 80%, 90%, 95%, and 100%) for 15 min for each elution. The samples were then lyophilized and coated with a palladium coater (Bal-TEC, Manchester, NH, USA). Each measurement was taken five times and the best image was selected.

### 2.11. Free Amino Acids (FAAs) Determination

A 2.00 g fish sample and 10 mL of 5% trichloroacetic acid were centrifuged at 10,000× *g* for 10 min. The extraction and centrifugation were repeated and the combination was diluted to 25 mL. 1 mL of the extract was filtered through a 0.22 μm disposable filter and assessed using an amino acid analyzer (Hitachi L-8800, Tokyo, Japan). Only one trial was undertaken per experiment.

### 2.12. Statistical Analysis

The data were analyzed via one-way ANOVA using SPSS 23.0 followed by Duncan’s test, and the results were expressed as the means ± standard deviation. The significance level was 0.05.

## 3. Results and Discussion

### 3.1. Thawing Rates and Curves

The thawing process of frozen foods is an essential factor in retaining their qualities, such as textural properties and color [25]. The RT treatment had the longest thawing time compared with the other treatments (Figure 2), and reached 64 h. The thawing time of the WT treatment was 70 min, and SUAT-28, SUAT-40, DUAT-28/40 took 45 min, 32 min, and 28 min, respectively. The UAT treatments were much faster, especially DUAT-28/40. The vibrational energy was attracted by the sample; thus, the frozen part of the sample quickly passed through the maximum ice crystal zone [26]. Among the three UAT treatments, DUAT was faster than SUAT, which was due to the cavitation effects produced by different ultrasound frequencies with mass transfer [1,27]. From −18 °C to −5 °C, the thawing rate was rapid and the central temperature rose sharply due to the difference between the sample temperature and the environmental temperature. However, from −5 °C to 0 °C, which was in the period of phase change of water in food, the thawing rate decreased, and the central temperature rose slowly [28]. The duration of this process is particularly important for the quality of frozen foods, and the UAT treatment significantly reduced the thawing time. Some studies have also indicated that UAT treatments shortened the thawing time of silver carp surimi [11].

### 3.2. Water Retention

Water retention is an important quality parameter that affects the texture, appearance, and storage stability of aquatic products [29]. Table 1 shows that the DUAT-28/40 treatment had the lowest thawing loss (1.70%) and the highest water-holding capacity (81.49%). However, there was no significant difference between the DUAT and WT samples in terms of the cooking loss (*p* > 0.05), which may be because the DUAT-28/40 treatment produced uniform force and internal stresses on the ice crystals of samples, resulting in less damage to cells from ice crystals. The increase in the thawing loss not only reduces the product weight, but also affects the product quality and eventually causes economic loss. Cooking loss includes large amounts of liquid and small amounts of soluble material from fish [30], which reflects structural damage to the muscle because of heat-induced denaturation of myofibrillar proteins. Therefore, the DUAT-28/40 treatment could better maintain water during the thawing process.

### 3.3. Texture and Color

Texture is related to physical properties and affects the sensory and functional characteristics of fish [31]. The hardness, springiness, chewiness, and resilience values of the samples which underwent DUAT-28/40 treatment were all higher than those of the other samples (Table 2). There were no significant differences between the SUAT-28 treatment and the SUAT-40 treatment (*p* > 0.05). This result was similar to the results of another study [5]. It was also determined that the cavitation effect generated by ultrasonic action possibly modified the mechanical radiation, to some extent. Because different thawing methods had different effects on the changes in the water retention and protein denaturation in fish meat, the changes in the texture characteristics of fish meat were also different [32,33,34]. This could also be explained by the fact that the radius of the bubbles in the DUAT field was greater than the radius of the bubbles in the SUAT fields. Under the same UAT time, the DUAT treatment produced a more pronounced cavitation effect than SUAT [1]. In addition, the ice crystals also caused different degrees of damage to tissue. Irregular ice crystals in the WT and RT treatments caused more damage to meat than those in the UAT treatments. There are two main reasons for the rapid thawing assisted by ultrasonic technology: first of all, the attenuation of ultrasound in the medium produces high-frequency oscillations that are converted to thermal energy [33], which could accelerate melting of ice crystals; in addition, ultrasonic treatment could produce cavitation and microstreaming during the UAT process. The cavitation bubbles could be used as the primary ice nuclei and the mechanical force generated by the bursting of the cavitation bubbles breaks the ice crystals into smaller pieces [33], which affects the ice morphology and formation.

Consumers often subjectively judge the quality of fish by its color, which is the external expression of the physiological, biochemical, and microbiological changes of the fish [35] One of the most commonly used indicators is L* (brightness/darkness). The L* value of the DUAT-28/40 treatment was higher than that of the other treatments, and there were no significant differences between the WT treatment and the DUAT-28/40 treatment (Table 2). Li et al. [6] found that the L* values of UAT treatments were higher than those of the control, which was the same as the result of this research. There were two possible mechanisms mentioned in the article for changes in the color values caused by UAT treatment: (1) the production of free radicals to promote oxidation, resulting in destabilization of heme pigments, and (2) possible changes in the chemical structure of myoglobin and heme pigments due to the thermic and acoustic influence of ultrasound [36]. This theory could explain the phenomenon in the study. Moreover, the formation of extracellular space was also indicated as a main reason for the color change in a study [37].

### 3.4. pH Value

**Figure 3:**
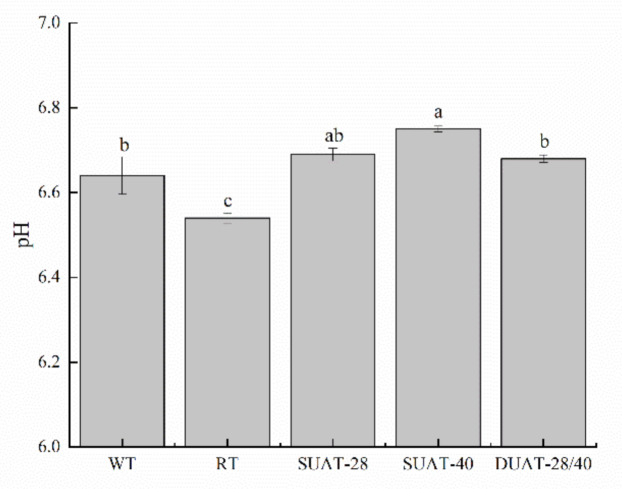
Differences in pH value of large yellow croaker under different thawing methods. (WT: water thawing; RT: refrigerated thawing; SUAT-28: single-frequency ultrasound-assisted thawing at 28 kHz; SUAT-40: single-frequency ultrasound-assisted thawing at 40 kHz; DUAT-28/40: dual-frequency ultrasound-assisted thawing at 28 kHz and 40 kHz). Different superscript letters indicate significant difference (*p* < 0.05).

The pH value is one of the indicators used to evaluate the freshness of fish samples, and it also has a great influence on the protein properties of fish [38]. The pH range of all treatments was 6.5–6.8, where the SUAT-40 treatment was highest, and the RT treatment was lowest (Figure 3). Ultrasound treatment might have damaged the cells, leading to endolysis. Nitrogenous substances decompose to alkaline substances, resulting in higher pH. The DUAT-28/40 treatment was not significantly different from the WT treatment (*p* > 0.05). Studies have shown that the decrease in the pH value is due to an increase in the solute concentration caused by the loss of water and the release of hydrogen ions [39]; this affected pH the most. A decrease in pH might have led to changes in the muscle structure which reduced its water retention. However, it was determined that pH was not the main factor leading to changes in quality [40].

### 3.5. Analysis of TVB-N and the K Value

**Figure 4:**
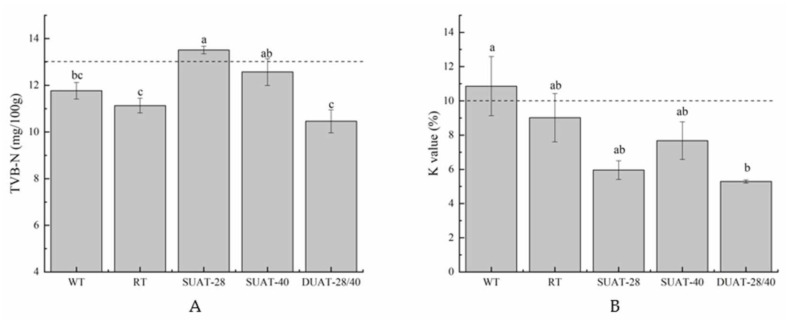
Differences in TVB-N value (mg/100 g) (**A**) and K value (%) (**B**) of large yellow croaker under different thawing methods. (WT: water thawing; RT: refrigerated thawing; SUAT-28: single-frequency ultrasound-assisted thawing at 28 kHz; SUAT-40: single-frequency ultrasound-assisted thawing at 40 kHz; DUAT-28/40: dual-frequency ultrasound-assisted thawing at 28 kHz and 40 kHz). Different superscript letters indicate significant difference (*p* < 0.05).

TVB-N generally increases with a decrease in freshness, so it is widely used as an important indicator to reflect the freshness of fish [41,42]. The DUAT-28/40 treatment had the lowest value at 10.46 mg/100 g (Figure 4A), which was still at the first-grade freshness standard [23]. This result indicated that fish spoilage was minimal with this thawing method [1]. In addition, there were no significant differences between the DUAT-28/40 treatment group and two controls (WT and RT) (*p* > 0.05). The results of TVB-N were better under the DUAT-28/40 treatment.

The K value is a relative value based on the quantification of ATP and its decomposition products, reflecting the degree of ATP degradation reaction after death. Moreover, it is one of the indices used to evaluate the freshness of fish [43]. The K value of the DUAT-28/40 treatment was 5.29% (Figure 4B), which was still at the first-grade freshness standard and similar to the result for the TVB-N. The same trend was found for the K value and TVB-N in another study, which was consistent with the results of this research [1]. From both of these sets of results it could be concluded that the DUAT-28/40 treatment showed a better freshness. Moreover, there were no significant differences between the sample which underwent the DUAT-28/40 treatment and the other samples, except the sample which underwent WT (*p* > 0.05). There was no direct explanation for the change in the K value under the ultrasound treatment. It was hypothesized that the reason for the lower K value was due to lower damage to muscle tissue. The K value was related to ATP degradation. The regular and small ice crystals under ultrasound treatment were less destructive to the tissue and delayed the degradation of ATP.

### 3.6. Analysis via LF-NMR

**Figure 5:**
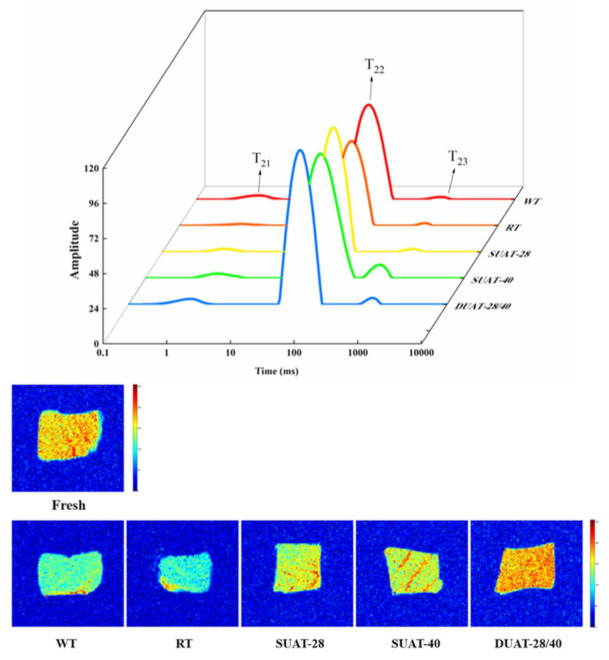
Differences in water distribution and magnetic resonance imaging of large yellow croaker under different thawing methods and in fresh sample. (WT: water thawing; RT: refrigerated thawing; SUAT-28: single-frequency ultrasound-assisted thawing at 28 kHz; SUAT-40: single-frequency ultrasound-assisted thawing at 40 kHz; DUAT-28/40: dual-frequency ultrasound-assisted thawing at 28 kHz and 40 kHz).

LF-NMR and magnetic resonance imaging (MRI) can be used to evaluate the distribution of water and the mobility of the muscle to learn about water migration due to different freezing treatments [44]. There are three peaks corresponding to the three relaxation components, named T_21_ (<10 ms), T_22_ (20–400 ms), and T_23_ (>1000 ms), which represent bound water, fixed water, and free water, respectively [24]. The peak of the samples changed under different thawing conditions, indicating that the thawing methods influenced the distribution of water in the muscle of fish. The DUAT-28/40 treatment had the highest T_21_ and T_22_ values (Figure 5). The high peak of bound water showed that a high level of bound water could be maintained and the damage to the fish was minimized. It also indicated that its combining ability was the strongest and had the lowest transfer of fixed water to free water, possessing a better water-migration effect and quality [1]. Studies have shown that the redistribution of ice crystals and mechanical damage are the root causes of water transfer and loss [45]. Irregular and large ice crystals disrupted the cell structure, leading to the release of cell contents and facilitating protein denaturation. Under UAT treatment, the ice crystals were small and uniform to avoid this problem. Harnkarnsujarit et al. [46] also indicated that the ice formation also modified protein conformation and denaturation of protein in fish muscles. Thus, the bound water content of the samples that underwent WT and RT was lower than that in those that underwent the UAT treatment.

A brighter red color indicates a higher ^1^H proton density and a higher moisture mass fraction, and a blue color indicates a low moisture content. The change in image color can reflect the water distribution of fish samples [47]. The color of the samples that underwent DUAT-28/40 treatment was redder than that of those that underwent the other treatments (Figure 5), indicating that the DUAT-28/40 treatment retained higher moisture levels. The control treatment samples all showed as blue in MRI, reflecting the higher free water content, severe moisture loss, and quality damage.

### 3.7. Microstructure of the Fish Muscle

**Figure 6:**
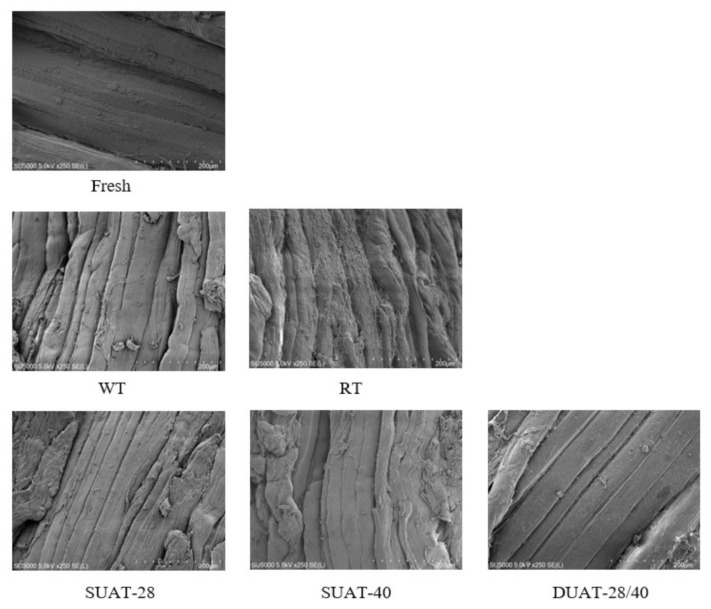
Differences in scanning electron microscope image of large yellow croaker under different thawing methods and fresh sample. (WT: water thawing; RT: refrigerated thawing; SUAT-28: single-frequency ultrasound-assisted thawing at 28 kHz; SUAT-40: single-frequency ultrasound-assisted thawing at 40 kHz; DUAT-28/40: dual-frequency ultrasound-assisted thawing at 28 kHz and 40 kHz).

Muscle microstructure is an important indicator of direct changes in muscle structural integrity. Studies have shown that muscle microstructure is related to water-holding capacity, texture, and myofibrillar protein properties [48]. As seen in Figure 6, the microstructure of the samples that underwent the WT and RT treatments was irregular and not flattened, and they were surrounded by adhesion on the surface. This result indicated that the bundles of myofibrils were severely damaged, resulting in protein denaturation, cell disruption, and damage to muscle structure [49]. The microstructure of the samples that underwent UAT was flatter, more regular, and smoother, especially after DUAT-28/40 treatment. Although the outer membrane exposed to the myofibrils was inevitably damaged, the overall integrity of the shape was essentially maintained. Irregular ice crystals damaged the structure of the muscles [50]. Under ultrasonic treatment, the ice crystals first melt into uniform and regular small ice crystals and then disappear slowly. The melting of ice crystals is an orderly process, which is consistent with the microstructure. Sun et al. [33] found that muscles treated with UAT-100 (UAT power at 100 W) and UAT-300 (UAT power at 300 W) had a more complete microstructure than the WT samples, with a slightly larger gap between the fascicles than the controls. Although the outer membrane exposed to the myofibrils was injured in the muscle, the inner membrane attached to myofibrils was relatively intact. This might be because the ultrasound decayed more rapidly in the unthawed region than in the thawed region, and the absorption of energy was mainly focused on the freeze–thaw border and the frozen part. This avoided the development of local overheating and thus decreased damage to the muscle bundle membrane. However, Li et al. [6] found that ultrasound treatment had little effect on the muscle tissue of fish.

### 3.8. Analysis of Free Amino Acids (FAAs)

**Figure 7:**
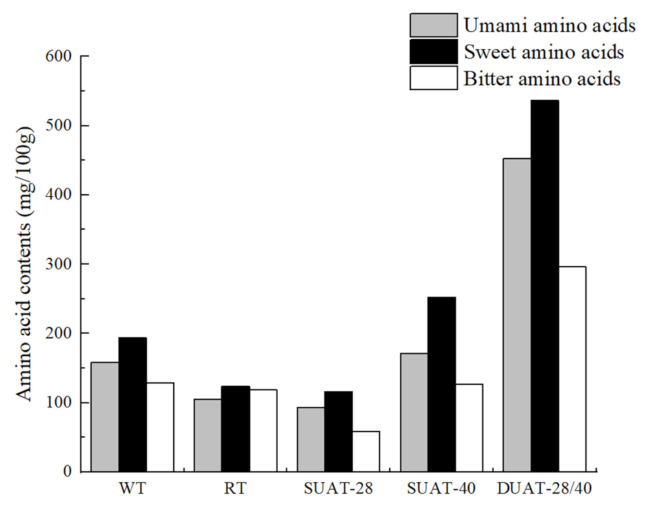
Differences in free amino acid contents (mg/100 g) of large yellow croaker under different thawing methods. (WT: water thawing; RT: refrigerated thawing; SUAT-28: single-frequency ultrasound-assisted thawing at 28 kHz; SUAT-40: single-frequency ultrasound-assisted thawing at 40 kHz; DUAT-28/40: dual-frequency ultrasound-assisted thawing at 28 kHz and 40 kHz).

The content of free amino acids is closely related to the flavor of fish [51]. Usually, there are three types of amino acids, including umami amino acids, sweet amino acids, and bitter amino acids. Among them, phenylalanine, tyrosine, lysine, and arginine play key roles, as they are the main biogenic amine precursors in fish [52]. As Table 3 shows, the highest contents of all FAAs were alanine, glycine, and lysine, and they played important roles in the taste and flavor of the fish. Kimbuathong et al. [53] also found that formation of amine compounds including trimethyl amine influenced the sensory characteristics of seafoods. Rich flavor amino acids contributed to improving the taste of fish. Alanine and glycine appeared to be umami and sweet, whereas lysine was bitter [54]. In terms of the total amino acids, the DUAT-28/40 treatment had the highest content, reaching 916.44 mg/100 g. This was obviously higher than that of the control, and DUAT increased the content of FAAs. The study by Kang [55] indicated that the cavitation caused by ultrasound could produce free radicals, which enhanced the level of protein degradation. The degradation of protein led to an increase in the FAA contents. In addition, it was discussed that high levels of ultrasound led to OH^-^ production from water molecules, which resulted in protein degradation and an increase in the FAA contents [56]. Among all the treatments, the sweet amino acids had the highest content, and the bitter amino acids were lower (Figure 7), which was related to the taste of the large yellow croaker [23]. The change in the taste presentation might be due to the degradation of ATP and associated compounds after fish spoilage, a loss of taste-presenting substances during thawing, and microbial metabolism. In summary, the cavitation effect of ultrasound in water could affect the degradation of proteins, which led to changes in the FAA contents. Moreover, Laorenza et al. [57] found that the enzymatic activity in seafoods caused the changes of protein conformation and degradation of protein into amino acids as well as degradation of lipid substances that influenced organoleptic qualities. The effect of the interaction between free amino acids and nucleotides on taste presentation should also be considered. Moreover, the Gly content was much higher in fish treated with DUAT-28/40 than other treatments. Because Gly is both an umami amino acid and a sweet amino acid, this phenomenon showed that the DUAT treatment contributed better to the flavor of fish.

## 4. Conclusions

In this experiment, we investigated the effects of single-frequency and dual-frequency ultrasonic treatment on the quality of large yellow croaker, including the thawing rate, water-holding capacity, and quality characteristics. Among all the thawing methods applied in the study, the DUAT-28/40 treatment was considered to be the best thawing method. The results showed that the UAT treatment significantly accelerated the thawing rate, and that the DUAT-28/40 treatment had the fastest thawing rate. It also had a better performance in terms of water-holding capacity, texture, color, and water distribution. Furthermore, the DUAT-28/40 treatment prevented the quality from being seriously damaged compared with other thawing methods in terms of the pH, TVB-N, and K values. It still stayed at the first-grade freshness standard under the DUAT-28/40 treatment. The microstructure analysis revealed less damage to the structure of the muscle under DUAT-28/40 treatment as the flatter, more regular, and smoother myofibrils showed. At the same time, the samples retained an impressive flavor, which was showed by the free amino acid results. Overall, the DUAT-28/40 treatment was a desirable thawing method for accelerating the thawing rate and improving the quality of large yellow croaker. Further research could explore the synergy between different ultrasound frequencies and power and how these apply in the thawing process. In addition, the mechanism is also expected to be studied.

## Figures and Tables

**Table 1:** Differences in thawing loss, water-holding capacity, and cooking loss of large yellow croaker under different thawing methods.

Treatments	Thawing Loss (%)	Water-Holding Capacity (%)	Cooking Loss (%)
WT	3.28	79.43 ± 3.21 ^a^	14.44 ± 2.48 ^c^
RT	1.97	75.08 ± 2.34 ^c^	26.88 ± 1.70 ^a^
SUAT-28	1.97	79.45 ± 2.76 ^a^	20.52 ± 1.13 ^b^
SUAT-40	3.22	77.58 ± 4.45 ^b^	21.42 ± 1.31 ^b^
DUAT-28/40	1.70	81.49 ± 2.19 ^a^	16.65 ± 1.40 ^bc^

WT: water thawing; RT: refrigerated thawing; SUAT-28: single-frequency ultrasound-assisted thawing at 28 kHz; SUAT-40: single-frequency ultrasound-assisted thawing at 40 kHz; DUAT-28/40: dual-frequency ultrasound-assisted thawing at 28 kHz and 40 kHz. Different superscript letters indicate significant difference (*p* < 0.05).

**Table 2:** Differences in hardness, springiness, chewiness, resilience, L*, a*, b*, and ΔE of large yellow croaker under different thawing methods.

Treatments	Hardness (g)	Springiness	Chewiness	Resilience	L*	a*	b*	ΔE
WT	2025.48 ± 68.43 ^b^	0.41 ± 0.03 ^b^	288.13 ± 48.37 ^ab^	0.137 ± 0.02 ^ab^	47.31 ± 1.54 ^ab^	0.02 ± 0.19 ^c^	−1.67 ± 0.39 ^c^	47.35 ± 1.53 ^ab^
RT	1891.09 ± 61.10 ^b^	0.44 ± 0.01 ^ab^	222.66 ± 27.78 ^bc^	0.093 ± 0.01 ^c^	43.39 ± 0.96 ^c^	0.28 ± 0.05 ^bc^	0.05 ± 0.22 ^a^	43.39 ± 0.96 ^c^
SUAT-28	1067.77 ± 43.34 ^c^	0.43 ± 0.01 ^ab^	185.64 ± 22.73 ^c^	0.114 ± 0.01 ^bc^	45.02 ± 1.23 ^bc^	0.57 ± 0.01 ^b^	−1.66 ± 0.45 ^c^	45.06 ± 1.21 ^bc^
SUAT-40	1427.11 ± 24.13 ^c^	0.41 ± 0.01 ^b^	262.31 ± 12.69 ^abc^	0.103 ± 0.01 ^c^	42.95 ± 0.63 ^c^	0.72 ± 0.43 ^a^	−1.68 ± 0.56 ^c^	43.00 ± 0.63 ^c^
DUAT-28/40	2482.59 ± 37.21 ^a^	0.47 ± 0.01 ^a^	349.69 ± 35.53 ^a^	0.152 ± 0.01 ^a^	49.80 ± 0.75 ^a^	0.72 ± 0.64 ^a^	−0.78 ± 0.89 ^b^	49.84 ± 0.74 ^a^

WT: water thawing; RT: refrigerated thawing; SUAT-28: single-frequency ultrasound-assisted thawing at 28 kHz; SUAT-40: single-frequency ultrasound-assisted thawing at 40 kHz; DUAT-28/40: dual-frequency ultrasound-assisted thawing at 28 kHz and 40 kHz. Different superscript letters indicate significant difference (*p* < 0.05).

**Table 3:** Differences in free amino acids contents (mg/100 g) of large yellow croaker under different thawing methods.

Free Amino Acids	Presentation of Taste	Treatments				
		WT	RT	SUAT-28	SUAT-40	DUAT-28/40
Aspartic acid (Asp)	umami	10.56 ± 0.55	4.19 ± 0.10	5.27 ± 0.01	9.18 ± 0.32	13.12 ± 0.42
Threonine (Thr)	sweet	28.59 ± 0.16	12.59 ± 0.25	14.18 ± 0.17	26.31 ± 1.18	37.05 ± 0.42
Serine (Ser)	sweet	30.39 ± 0.30	19.96 ± 0.30	16.63 ± 0.12	55.25 ± 2.58	88.15 ± 2.65
Glutamic acid (Glu)	umami	29.95 ± 0.24	15.95 ± 0.29	7.48 ± 0.09	ND	64.67 ± 1.27
Glycine (Gly)	umami, sweet	38.17 ± 0.51	25.64 ± 0.46	22.50 ± 0.08	58.23 ± 2.29	237.45 ± 6.58
Alanine (Ala)	umami, sweet	80.07 ± 0.90	59.37 ± 1.11	57.93 ± 0.40	103.98 ± 4.59	137.14 ± 3.92
Cysteine (Cys)	-	2.00 ± 0.08	1.96 ± 0.09	1.43 ± 0.04	3.26 ± 0.17	6.08 ± 0.18
Valine (Val)	bitter	21.95 ± 0.29	10.81 ± 0.24	8.00 ± 0.10	8.86 ± 0.45	12.24 ± 0.27
Methionine (Met)	bitter	10.17 ± 0.10	7.64 ± 0.19	6.68 ± 0.19	6.08 ± 0.39	9.33 ± 0.16
Isoleucine (Ile)	bitter	12.76 ± 0.15	6.29 ± 0.11	4.92 ± 0.06	4.76 ± 0.21	6.72 ± 0.19
Leucine (Leu)	bitter	23.35 ± 0.25	11.71 ± 0.27	9.19 ± 0.06	9.56 ± 0.42	12.54 ± 0.25
Tyrosine (Tyr)	bitter	9.64 ± 0.04	6.26 ± 0.22	6.90 ± 0.09	6.21 ± 0.32	8.67 ± 0.24
Phenylalanine (Phe)	bitter	9.06 ± 0.16	6.55 ± 0.20	5.53 ± 0.10	5.34 ± 0.29	7.72 ± 0.25
Lysine (Lys)	bitter	29.71 ± 0.35	55.77 ± 1.35	8.46 ± 0.16	52.37 ± 2.82	171.82 ± 3.74
Histidine (His)	bitter	12.56 ± 0.19	13.40 ± 0.33	9.41 ± 0.08	34.10 ± 1.81	67.18 ± 1.57
Arginine (Arg)	sweet	0.66 ± 0.02	1.59 ± 0.07	0.04 ± 0.04	2.38 ± 0.18	2.63 ± 0.00
Proline (Pro)	sweet	15.82 ± 5.45	4.42 ± 1.07	5.13 ± 0.01	6.25 ± 1.04	33.93 ± 11.13
Total		365.40 ± 9.73	264.07 ± 6.66	189.66 ± 1.50	392.12 ± 19.04	916.44 ± 33.23

WT: water thawing; RT: refrigerated thawing; SUAT-28: single-frequency ultrasound-assisted thawing at 28 kHz; SUAT-40: single-frequency ultrasound-assisted thawing at 40 kHz; DUAT-28/40: dual-frequency ultrasound-assisted thawing at 28 kHz and 40 kHz.

## Data Availability

Not applicable.
